# Exploring the spiking neural autoencoder: from hyperexcitability to noise-driven compensation

**DOI:** 10.3389/fnsys.2026.1788937

**Published:** 2026-06-04

**Authors:** Mohammadreza Khodashenas, Daniel P. Martins

**Affiliations:** 1 Walton Institute for Information Systems Science, Department of Computing and Math, South East Technological University, Waterford, Ireland; 2 Department of Engineering Technology, South East Technological University, Waterford, Ireland; 3 FutureNeuro Research Ireland Centre, Dublin, Ireland; 4 School of Computer Science and Electronic Engineering, University of Essex, Colchester, United Kingdom

**Keywords:** autoencoder, biological neural modeling, computational neuroscience, Gaussian noise, image compression, LIF neuron model, spiking neural network, temporal lobe epilepsy

## Abstract

**Introduction:**

Understanding how artificial neural networks (ANNs) can capture biologically meaningful dynamics is a central challenge in systems neuroscience. In this work, we investigate whether spiking neural networks (SNNs) can function not only as machine-learning tools but also as biologically inspired computational analogs and tractable *testbeds* for studying pathological neural dynamics.

**Methods:**

We implemented a spiking autoencoder composed of Leaky Integrate-and-Fire and Synaptic neuron models to create a controlled framework for analyzing how biologically related parametric changes to neuronal and synaptic dynamics influence learning and information transfer. By tuning model parameters to induce persistent overfiring-like behavior, we emulated a hyperexcitability-like regime conceptually analogous to NaV channel dysfunction in hippocampal circuits. Reconstruction performance and network activity were evaluated under both noiseless and noisy conditions.

**Results:**

The induced hyperexcitability-like regime degraded image reconstruction performance and disrupted stable information propagation, consistent with impaired processing in hyperexcitable neural systems. Layer-wise firing-rate analysis revealed that the altered regime was characterized by unstable activity redistribution rather than sustained global overactivation. Importantly, introducing controlled Gaussian noise into the input stream partially restored reconstruction quality and improved learning performance, suggesting that stochastic perturbations can partially compensate for instability in dysfunctional network regimes.

**Discussion:**

These findings demonstrate that specific SNN parameter regimes can reproduce key signatures of pathological excitability while also providing a platform for investigating compensatory mechanisms. Overall, this work positions spiking autoencoders as scalable, biologically grounded frameworks for hypothesis-driven studies of neural dysfunction and candidate interventions, supporting the integration of ANN methodologies with mechanistic models in systems neuroscience.

## 1 Introduction

Artificial intelligence (AI) traces many of its core ideas to brain physiology, inspiring the design of neural-network algorithms that approximate how biological systems process information. Over the past decades, a range of architectures has been developed, including convolutional neural networks (LeCun et al., 1989, 1998), recurrent neural networks (Graves et al., 2013), and graph neural networks (Wu et al., 2020). Beyond software, neuromorphic hardware has emerged to emulate neuronal computation in silicon (Upadhyay et al., 2019; Zhao et al., 2023).

While artificial neural networks (ANNs) are typically optimized as engineering tools, spiking neural networks (SNNs) occupy a distinctive niche: by explicitly modeling spike timing and synaptic dynamics, they provide an intermediate framework that is both computationally scalable and grounded in biologically meaningful parameters (Guo et al., 2023). SNNs capture temporal dynamics and spike-based information processing observed in biological neural systems, and have been applied across tasks such as ECG classification and radar-based action recognition (Chu et al., 2022; Fontanini et al., 2022). Crucially, because SNNs expose parameters that map onto neuronal and synaptic physiology, it seems they have a potential capability to be used not only to solve tasks but also as tractable, hypothesis-driven platforms for studying network dynamics that are relevant to systems neuroscience.

The brain learning process plays a fundamental role in cognitive function and knowledge acquisition (Conway, 2020). Motivated by this, we adopt the perspective that SNNs can serve as *biological network analogs* to probe how altered neuronal excitability affects network-level computation and learning (Botvinick et al., 2020). In particular, we examine whether parametric changes to neuron and synapse dynamics in an SNN can represent abnormalities analogous to those arising from NaV channel dysfunction in hippocampal circuits (e.g., hyperexcitability implicated in Temporal Lobe Epilepsy), and whether simple interventions can mitigate those effects.

To provide a controlled experimental scaffold, we implement a spiking autoencoder that performs image compression on handwritten digit images. Autoencoders compress high-dimensional inputs into a lower-dimensional latent representation and then reconstruct approximations of the original data (Theis et al., 2017). Here, the autoencoder is used as a diagnostic task: it exposes how changes in neuronal and synaptic parameters (e.g., those that increase spiking rate) might influence information encoding, transport, and decoding across network layers.

Concretely, we design the autoencoder using a combination of Synaptic and modified Leaky Integrate-and-Fire (LIF) neuron models and ask the following question: *Can neurons and networks parameterized to overfire sustain learning and processing performance comparable to networks operating under biologically typical excitability?* We investigate both noiseless and noisy inputs (Gaussian noise) to test whether stochastic perturbations can stabilize network dynamics under emulated pathological-like regimes. In this work, SNNs are not evaluated as direct competitors to conventional artificial neural networks in terms of performance metrics. Instead, they are employed as computational testbeds to investigate how controlled changes in neuronal and synaptic dynamics influence network-level behavior under different dynamical regimes.

Our contributions are therefore threefold: (1) we demonstrate that tuning SNN parameters to induce overfiring-like reproduces degraded information processing consistent with hyperexcitable biological circuits; (2) we show that adding Gaussian noise has the potential to counteract network instability, or even might act as a stabilizing intervention, that improves learning and reconstruction in otherwise unstable regimes; and (3) we position SNN-based autoencoders as tractable platforms for hypothesis testing at the interface of ANN methodologies and mechanistic models of neuronal dysfunction, thus advancing the goals of the “Unraveling Neural Network Dynamics” Research Topic.

## 2 Materials and methods

The methodological framework presented below is designed to operationalize the central objective of this study: using spiking neural networks as a controlled computational testbed for investigating neural network dynamics under physiological-like and hyperexcitable-like regimes. Rather than optimizing performance alone, the selected neuron models, parameters, and task are explicitly chosen to emulate learning, information propagation, and instability mechanisms relevant to hippocampus-related hyperexcitability and noise-driven compensation.

SNNs are inspired by natural neural communication processes and use an artificial representation of time-dependent spikes to propagate information (Rathi et al., 2023). In such networks, the information is only transmitted when the neuron membrane potential exceeds a certain threshold instead of on each propagation cycle, like in typical artificial neural networks (Kramer, 1991). Different methods have been explored to model the spike activation in artificial neurons, resulting in several neuronal network architectures that are able to solve machine learning problems, such as data classification and pattern recognition (Guo et al., 2023). Traditionally these applications use SNNs as task-oriented tools; here we emphasize their alternate role as biological-network analogs and tractable testbeds for studying neural dynamics.

**Figure 1:**
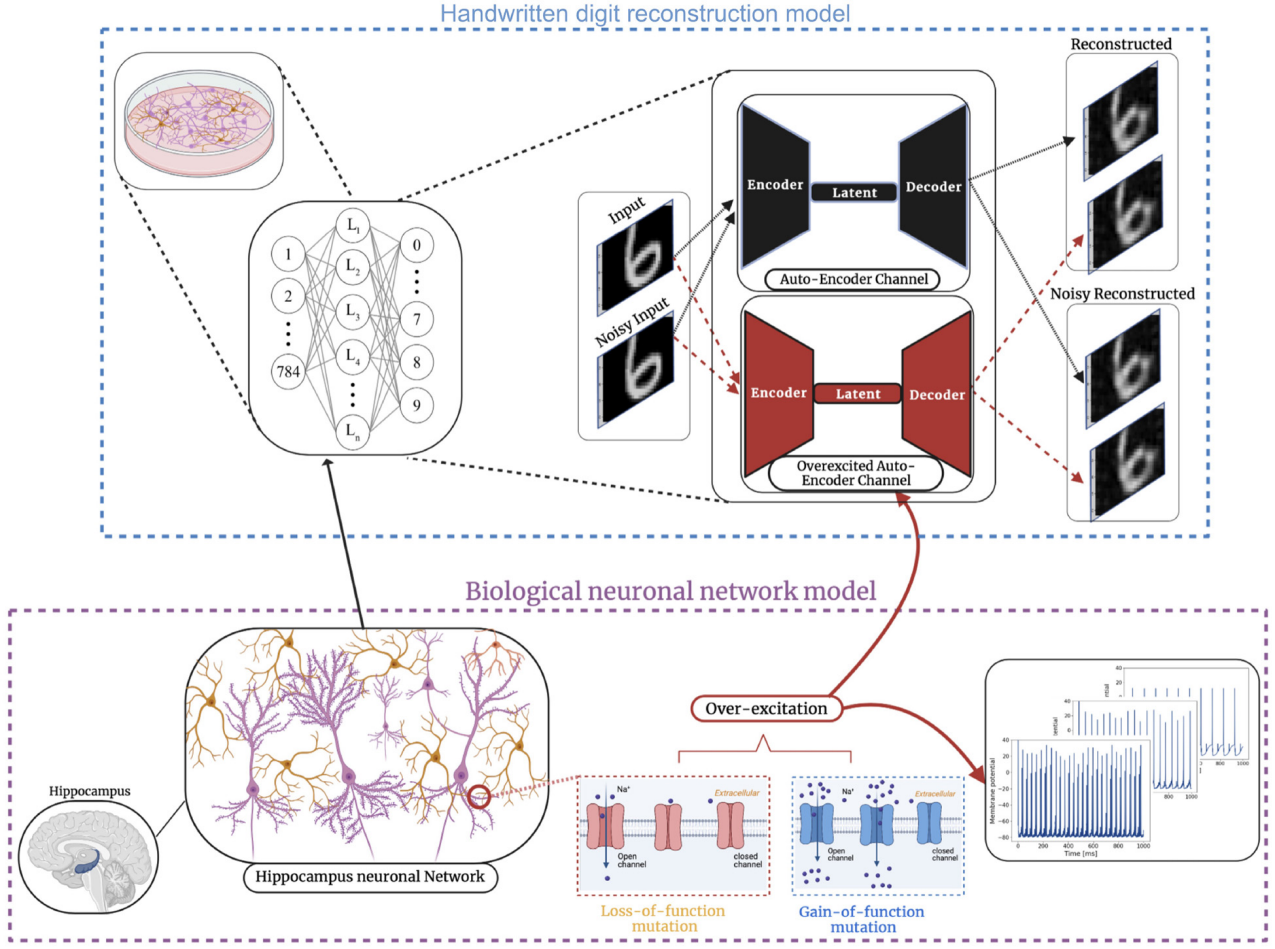
Schematic of autoencoder architecture related to hippocampus neuronal network and their related equivalent electrical circuit to observe the effect of overfiring-like on an image data recognition learning process.

Our SNN (see Figure 1) expands the architectures proposed in the literature by combining LIF and Synaptic neuron models, allowing us to control the generation of spikes and observe their effect on the network performance while processing handwritten digit images.

The LIF model is widely used in neural networks due to its lower complexity and higher computational efficiency compared to more complex biophysical models,such as the Hodgkin–Huxley (HH) model (Eshraghian et al., 2023). Although the HH model describes the influence of the ionic channel on the action potentials of a neuron in detail, the LIF model provides a spike pattern, which is useful when investigating time-dependent or spiking behavior of neurons (Börgers, 2017). The LIF model lies between conventional Artificial Neural Networks, an abstract representation that is practical toward AI applications, and the HH model, which is oriented toward biological realism. In other words, the LIF model occupies an intermediate modeling regime: more biologically grounded than typical ANNs yet more scalable than full biophysical models such as Hodgkin–Huxley.

**Figure 2:**
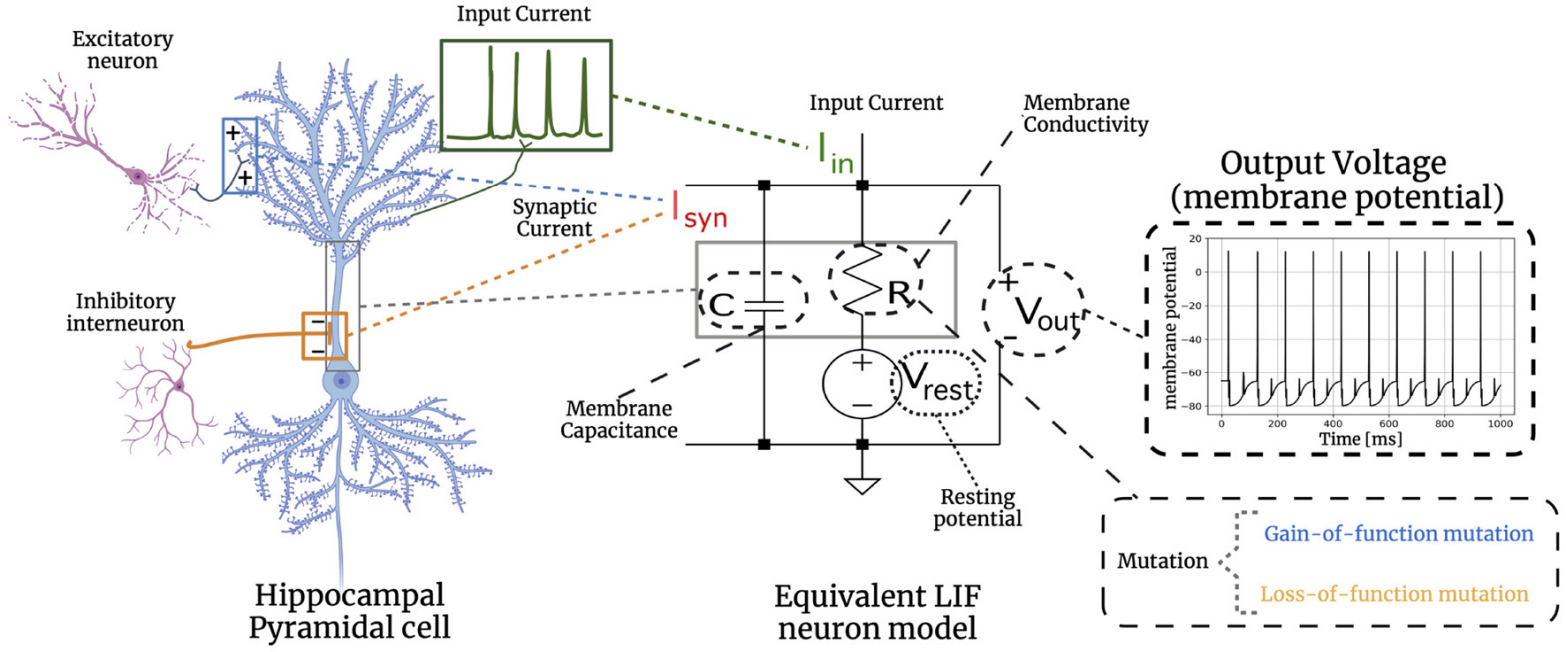
LIF Neuron Model as a parallel resistor capacitor (RC) circuit; This model captures the essential dynamics of neuronal spiking by simulating the integration of synaptic inputs (*I*_*syn*_) and the subsequent generation of action potentials. It also shows how the Gain/Loss of function mutations can be associated with the resistance (conductivity) component, ultimately affecting the membrane potential.

In the LIF model, a neuron is represented by an electronic circuit that includes a capacitor and a resistor in parallel, driven by an input current, as shown in Figure 2. The membrane potential *V*_*out*_ is the activation function for this neuron model and is evaluated considering its input current *I*_*in*_, the resistance *R*_*mem*_ and capacitance *C*_*mem*_ of the membrane (Gerstner et al., 2014). Therefore,


$$\begin{array}{l} V_{out}\left(t\right)=R_{mem}\times I_{in}\left(t\right)+\left[V_{0}-R_{mem}\times I_{in}\left(t\right)\right]e^{-\frac{t}{\tau }} \end{array}$$ (1)

where τ = *R*_*mem*_*C*_*mem*_ and it represents the time taken by the membrane potential to reach a certain percentage of its steady-state value. By solving (Equation 1) when the input current is equal to zero, *I*_*in*_(*t*) = 0, and Δ*t* < < τ (Eshraghian et al., 2023), we obtain


$$\begin{array}{l} \frac{V\left(t+\Delta t\right)}{V\left(t\right)}=\left[1-\frac{\Delta t}{\tau }\right]=\beta \end{array}$$ (2)

where β is the LIF neuron decay rate. Here, we use β to increase/decrease the generation of spikes in the LIF model and observe its relation with the neuron conductivity, *g*, by assessing (Equation 2) in terms of the initial state of the neuronal network (that is, when *I*_*in*_(*t*) = 0 and *V*_*out*_(*t*) = *V*_0_) as follows (Equation 3):


$$\begin{array}{l} \beta =\frac{\left[V_{0}\right]e^{-\frac{t+\Delta t}{\tau }}}{\left[V_{0}\right]e^{-\frac{t}{\tau }}}=e^{-\frac{\Delta t}{\tau }}=e^{-g\frac{\Delta t}{C_{mem}}}, \end{array}$$ (3)

then we consider Δ*t* = *C*_*mem*_ to obtain


$$\begin{array}{l} \beta =e^{-g}, \end{array}$$ (4)

which produces similar values to the ones typically measured in healthy biological neurons (e.g., *g* = 0.1 mS results in β≈0.9) (Khodashenas et al., 2019; Eshraghian et al., 2023). From (Equation 4), we can conclude that lower β values, results in higher *g* values which is related to neuronal conductivity; in this work, the term overfiring-like and/or hyperexcitability-like regime is defined as increased neuronal conductivity *g*, which is now mapped to reduced decay parameter β in the LIF model. This parameterization is inspired by channel/leak conductivity increases in membrane excitability in biological neurons.

**Figure 3:**
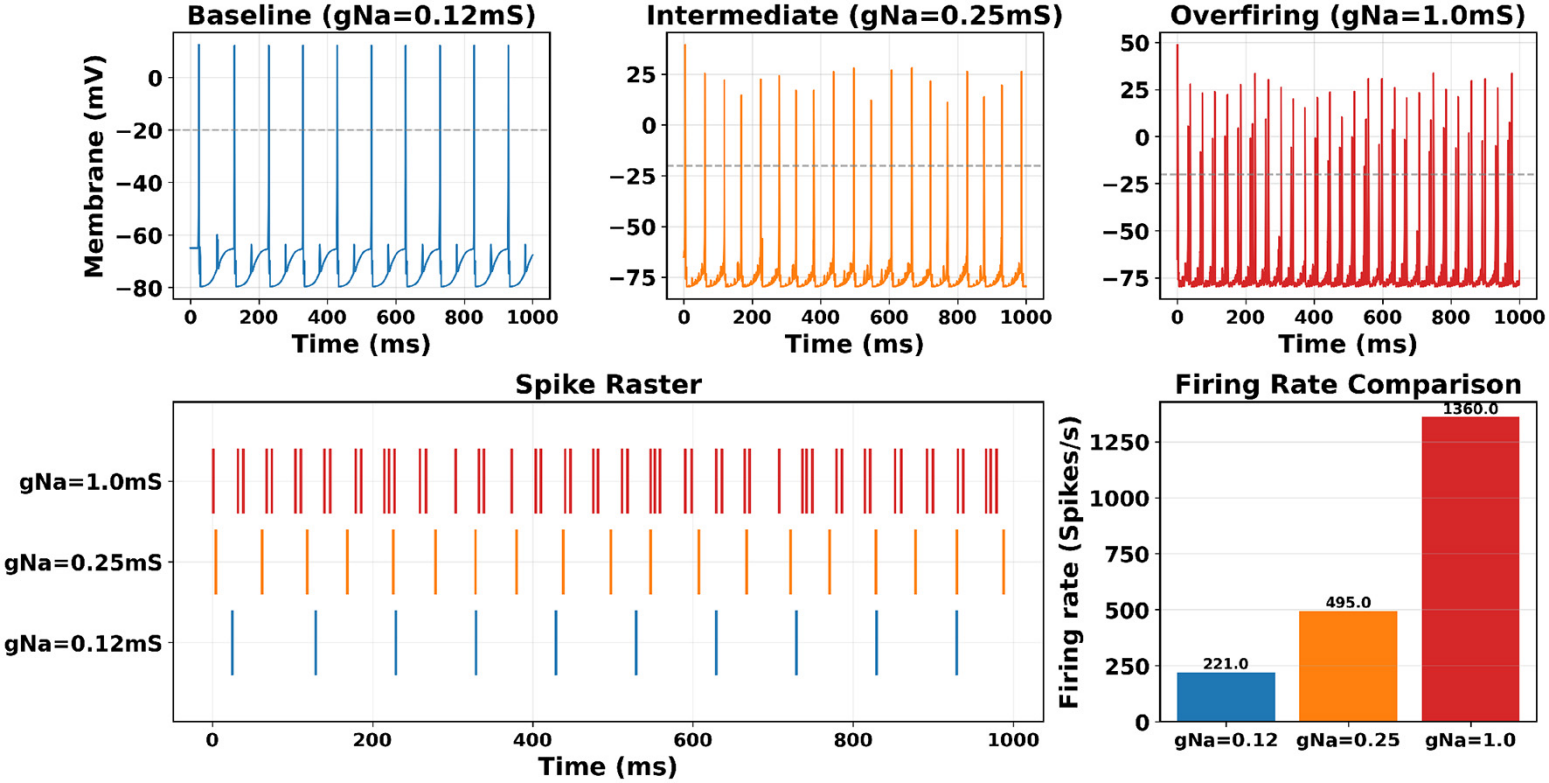
Spike counts, firing rates, and membrane potential dynamics based on three levels of neuronal conductivity *g*_*Na*_ = 0.12*mS* as, *g*_*Na*_ = 0.25*mS* as *intermediate*, and *g*_*Na*_ = 1.0*mS* as *overfiring/hyperexcitable*.

Regarding the biological neurons behavior, we inducate hyperexcitability evidence, using direct electrophysiological measurements obtained from the NEURON simulator in Figure 3. This biophysical overfiring condition has been shown using increased *Na*_*V*_ voltage-gated sodium channel conductivity (*g*_*Na*_) results in a marked increase in spike rate and reduced temporal stability compared to baseline conductance regimes, confirming a mechanistic hyperexcitability phenotype that supports the hypothesis based on increased neuronal conductivity. This neuronal dynamic has been quantified not only by membrane potential dynamics but also including spike counts/raster and firing rate comparison for different (*g*_*Na*_). Three different status has been shown, *g*_*Na*_ = 0.12 mS for condition, *g*_*Na*_ = 0.25 mS as intermediate status, and finally *g*_*Na*_ = 1.0 mS as overfiring/hyperexcitable regime.

For a defined β value, the SNN will then optimize its performance by updating its weights (filter or kernel), using the loss gradient (Eshraghian et al., 2023), while executing the image compression task; critically, we use the compression task as a controlled scaffold to probe how altered excitability impacts network-level computation rather than as an end in itself. This learning process is given by solving (Equation 1), using a forward Euler method, when

*R*_*mem*_ = 1 (reducing the losses that may occur by triggering the SNN), resulting in the following activation function


$$\begin{array}{l} V_{out}\left(t\right)=\left[1-W_{1}\right]V\left(t-1\right)+\left[W_{1}\right]X\left(t\right)-r_{\text{(reset)}}, \end{array}$$ (5)

where *W*_1_ = Δ*t*/τ is the learnable weight, *X*(*t*) is the network input, and *r*_(reset)_ = *V*_0_(Δ*t*/τ) allows to reset the network behavior whenever a spike is output from the neuron. In other words, to minimize (Equation 5), the network calculates the loss gradient for each learnable parameter by applying the chain rule from the final layer back to each weight, leading to an error reduction on the image compression task.

For the proposed SNN-based autoencoder, we also modulate the spike generation through the synaptic conductance. Natural neurons regulate their communication by activating their AMPA and NMDA receptors facilitating the passage of potassium and sodium ions, which can result in an increased generation of spikes, and are involved in various neurological functions and disorders (Paoletti et al., 2013). In practical terms, the Synaptic neuron model implements temporally extended synaptic currents (parameterized by α and β_*syn*_), enabling us to manipulate synaptic decay and thereby emulate synaptic dynamics linked to pathological hyperexcitability. From a biological perspective, when using the Synaptic neuron model we are gradually releasing neurotransmitters from the pre- to post-synaptic cell, instead of an using the instantaneous jump in the synaptic current. To observe this gradual temporal dynamics of the input current, we modify (Equation 2) to include the synaptic current and solve it using a forward Euler method, which results in Equation 6:


$$\begin{array}{l} V_{out}\left(t+1\right)=\beta_{\text{syn}}V_{out}\left(t\right)+{\text{I}}_{\text{syn}}\left(t+1\right)-r_{\text{(reset)}}\\ {\text{I}}_{\text{syn}}\left(t+1\right)=\alpha {\text{I}}_{\text{syn}}\left(t\right)+W\cdot X\left(t+1\right) \end{array}$$ (6)

where α is the synaptic current decay, and β_syn_ is the synaptic decay rate.

### 2.1 Lossy Image Compression using SNNs

**Figure 4:**
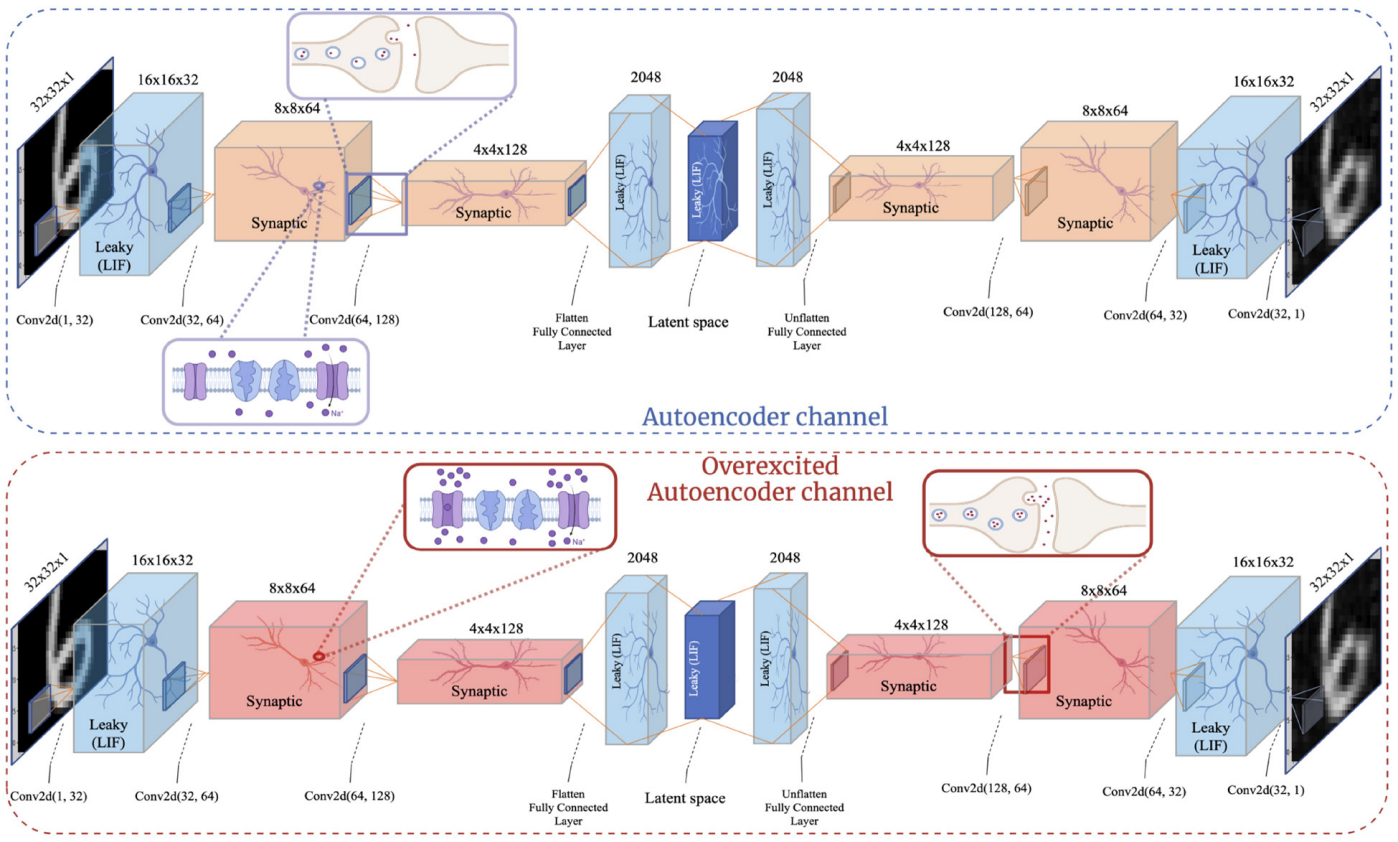
Network architecture of the autoencoder model based on LIF and Synaptic neuron models in encoder and decoder parts.

To investigate the behavior of neurons when subjected to an increased spiking rate, we created a SNN-based autoencoder, integrating LIF and Synaptic neuron models, capable of compressing handwritten digits. To this end, we used handwritten digit images from the MNIST dataset (Modified National Institute of Standards and Technology), which is an extensive collection of handwritten digits and is a popular resource for training various image processing systems (LeCun et al., 1998). To compress these image data, we opt to design an autoencoder, which has been applied to compress images (with losses) in unsupervised learning scenarios (Kramer, 1991; Takeishi and Kalousis, 2021). In the proposed architecture, the LIF and Synaptic neuron models are layered in a specific order to encode, transport, decode, and reconstruct handwritten digit images. The proposed architecture is shown in Figure 4, where the encoder performs a series of operations on the handwritten digit image to extract its compressed representation. Through these operations, the encoder progressively reduces the spatial dimensions and increases the number of channels in the data until a flattened representation (i.e., encoded latent space) is obtained. Then, an additional linear layer is used to transform the latent space back to the shape of the encoder's final layer. The decoder then performs inverse operations to reconstruct the original input data from the encoded handwritten digit images, resulting in a compressed version of the original input data (see Table 1 for the autoencoder configuration).

**Table 1:** Configuration of the proposed autoencoder application for the proposed SNN.

Layers	Height	Width	Channels	Filter size	Neuron model
Original/Reconstructed image	32	32	1		
EN-Convolutional 1 (EC1)	16	16	32	3 × 3	LIF
EN-Convolutional 2 (EC2)	8	8	64	3 × 3	Synaptic
EN-Convolutional 3 (EC3)	4	4	128	3 × 3	Synaptic
EN-Linear Layer (Flatten)					LIF
LS-Linear Layer	–	–	128 × 4 × 4	–	LIF
DE-Linear Layer (unFlatten)					LIF
DE-Transposed Convolutional 1 (DC1)	4	4	128	3 × 3	Synaptic
DE-Transposed Convolutional 2 (DC2)	8	8	64	3 × 3	Synaptic
DE-Transposed Convolutional 3 (DC3)	16	16	32	3 × 3	LIF

Please note that EN is referring to encoder,LS to latent space,and DE to decoder.The inclusion of the Synaptic neuron model in the layers EC2,EC3,DC1 and DC2 aimed to replicate the temporal dynamics of biological neural systems

To compress an image using the SNN-based autoencoder, we first utilize a black and white handwritten digit image without noise (32 by 32 pixels) as the network input *X*(*t*), see Figure 4. Please note that noisy images can also be used as input to the proposed SNN-based autoencoder, and in Section 3.1 we add Gaussian noise to handwritten digit images and investigate the SNN's network performance under such conditions. The noise experiments are explicitly framed as interventions to probe noise-driven compensation in pathological parameter regimes. Then, we reduce the spatial dimensions of the input signal *X*(*t*) by convolving it with a kernel filter *K* (a weight 3 by 3 matrix) in the layer EC1 as follows:


$$\begin{array}{l} F\left(i,j\right)=\left(X*K\right)\left(i,j\right)=\overset{1}{\underset{m=-1}{\sum }}\overset{1}{\underset{n=-1}{\sum }}X\left(i+m,j+n\right)\cdot K\left(m+2,n+2\right)\\ =X\left(i-1,j-1\right)\cdot k_{1,1}+X\left(i-1,j\right)\cdot k_{1,2}+X\left(i-1,j+1\right)\cdot k_{1,3}\\ +X\left(i,j-1\right)\cdot k_{2,1}+X\left(i,j\right)\cdot k_{2,2}+X\left(i,j+1\right)\cdot k_{2,3}\\ +X\left(i+1,j-1\right)\cdot k_{3,1}+X\left(i+1,j\right)\cdot k_{3,2}+X\left(i+1,j+1\right)\cdot k_{3,3}, \end{array}$$ (7)

where *F*(*i, j*) is the output feature map as (X*K)(*i, j*) represents the value of the convolution at position (*i, j*), * denotes the convolution operation, *m* and *n* are indices that iterate over the dimensions of the kernel. Please note that a similar operation is performed for layers EC2 and EC3. In (Equation 7) we show how each pixel in the output feature map is computed by taking a weighted sum of its neighboring pixels in the input image, with the weights defined by the kernel. Using a 3 × 3 kernel is common practice in convolutional neural networks because it effectively captures local spatial patterns in the input image while keeping computational complexity manageable (Simonyan and Zisserman, 2014). We repeat this process twice using the Synaptic neuron model instead of the LIF. The final layer of the encoder is another SNN based on the LIF model, which flattens the handwritten digit image to be processed and transported to the decoder. Please note that we opted to have the LIF models at the input and output layers of the encoder to observe the impact of the synaptic current on the signal propagation through the encoding network and not on the information acquisition/retrieval. In other words, the focus is on how the signal (information) moves through the network rather than how the network retrieves or acquires information. Therefore, we are interested in investigating the effects of increased spiking rate on the signaling process of our SNN-based autoencoder, which may affect its learning capabilities. Please also note that the decoder does the reverse operations executed by the encoder, therefore, we use the same layer composition of the encoder.

#### 2.1.1 SNN-based autoencoder performance analysis

To achieve a good performance, all the autoencoder's SNNs have to learn how to adapt themselves to the variations the hyperparameters may suffer due to the scenarios that they are subjected to. This learning process occurs by updating the SNNs' weights through backpropagation and adapted gradient descent algorithms (i.e., surrogate gradient); see (Eshraghian et al., 2023; Lee et al., 2016; Neftci et al., 2019). After compressing the handwritten digits, we compute the mean square error to assess the performance of SNN. We define two experimental regimes, a biologically-informed “benchmark" (healthy-like excitability) and an “overfiring-like" (pathological/hyperexcitable) regime, and use metrics to quantify how these regimes affect encoding, latent representations, and reconstruction *MSE* is used to observe and quantify how much error occurs during the image compression task, and *E*_*l*_ to measure the level of uncertainty of each autoencoder's layer when performing the same task. It is worth mentioning that, the experimental design focuses on controlled manipulation of spiking dynamics rather than exhaustive benchmarking across architectures or hyperparameter spaces.

Here, we also measure the *MSE* displacement, *MSE*_*d*_, to observe how much these metrics vary over a set number of SNNs' iterations; and the layer's *MSE*, *MSE*_*l*_, when comparing the data difference between the scenario with a typical and the excessive spiking rates (hereafter named benchmark and overfiring-like scenarios, respectively). Please note that *MSE*_*l*_ is the only metric that applies a direct comparison between the scenarios we devised for this work. The remaining metrics are evaluated for each scenario separately.

The *MSE* measures the difference between the original and output data, and is used to trigger the computation of gradients for all the learnable parameters in the network through backpropagation, to update the weights of each SNN. Although this is not the focus of this work, it is important to note that *MSE* optimization can lead to improved SNN performance. Here, we focus on measuring data loss during the image compression task using *MSE* as an indicator of the network learning process. Therefore, we compute *MSE* as follows (Equation 8) (Weng, 2020),


$$\begin{array}{l} MSE=\frac{1}{n}\overset{n}{\underset{i=1}{\sum }}{\left(x_{i}-{\hat{x}}_{i}\right)}^{2}, \end{array}$$ (8)

where *x*_*i*_ is a handwritten data sample, *i* = 1, ..., *n* is the sample index, and ${\hat{x}}_{i}$ the reconstructed handwritten data sample. With a defined number of training and testing iterations of the SNNs, we evaluate the *MSE*_*d*_ as the area of the *MSE* over the number of iterations plot curve, which we obtain using a trapezoidal rule as follows (Equation 9) (Atkinson, 1991)


$$\begin{array}{l} MSE_{d}=\overset{m}{\underset{j=1}{\sum }}\frac{\left(MSE_{j}+MSE_{j-1}\right)}{2}\times \Delta j, \end{array}$$ (9)

where *MSE*_*j*_ is the *MSE* value at iteration *j*, Δ*j* is the difference between consecutive iterations, and *j* = 1, ...*m* is the iterations index. In this context, *MSE*_*d*_ quantifies the *MSE* behavior for the autoencoder's training and testing iterations. Then, we evaluate the *MSE*_*l*_ by quantifying the difference between corresponding elements of the benchmark and overfiring-like scenarios feature maps as follows (Equation 10):


$$\begin{array}{l} MSE_{l}=\frac{1}{H\cdot W}\overset{H}{\underset{i=1}{\sum }}\overset{W}{\underset{j=1}{\sum }}{\left({\text{B}}_{l}\left[i\right]\left[j\right]-{\text{O}}_{l}\left[i\right]\left[j\right]\right)}^{2} \end{array}$$ (10)

where *H* and *W* represent the height and width of the feature maps, respectively, *B* and *O* stand for the benchmark and overfiring-like scenarios, respectively. The *MSE*_*l*_ allows us to observe the data loss behavior for each layer of the autoencoder when subjected to a typical or excessive spiking rate.

Spike activity in the SNN was defined as the binary output of spiking neuron modules (0 or 1), corresponding to threshold-crossing events in Leaky Integrate-and-Fire (LIF) and Synaptic neuron models. Mean firing rate for each layer was computed as the average spike probability across all neurons, spatial locations, and time steps in Equation 11:


$$\begin{array}{l} FR_{\ell }=\frac{1}{NT}\overset{N}{\underset{i=1}{\sum }}\overset{T}{\underset{t=1}{\sum }}s_{i}^{\ell }\left(t\right), \end{array}$$ (11)

where $s_{i}^{\ell }\left(t\right)\in \left\{0,1\right\}$ denotes the spike output of neuron *i* in layer ℓ at time *t*, *N* is the number of neurons, and *T* is the number of simulation steps.

## 3 Results

The results are organized to examine how parametrically induced hyperexcitability alters network learning dynamics and information reconstruction, and how stochastic input perturbations (Gaussian noise) modulate these effects. We first characterize baseline (benchmark) and hyperexcitable-like (overfiring-like) regimes, then assess how noise influences stability, layer-wise information flow, and latent representations. To evaluate the information encoding and reconstruction performance of the proposed SNN-based autoencoder, we first designed our benchmark and overfiring-like scenarios. Then, we compressed noiseless and noisy MNIST-sourced handwritten digit images using our SNN-based autoencoder (considering both scenarios) and computed the data lost in this process using the metrics introduced in Section 2. For the benchmark scenario, we defined hyperparameter values that emulate the network behavior of biological neurons; β = 0.9, α = 0.9 and β_*syn*_ = 0.9 for the LIF and Synaptic neuron models (Eshraghian et al., 2023; Khodashenas et al., 2019). We considered a unitary threshold value for all neuron models (τ = 1) and set the number of steps as *num*_*step*_ = 5. We also defined the total number of epochs to 50 and utilized the AdamW optimizer (i.e., the widely used tool in deep learning for optimizing model parameters during training), setting the parameter *lr* to 0.0001, the values of the parameters *betas* of (0.9, 0.999) and *weight*_*decay*_ = 0.001 (Loshchilov and Hutter, 2017). We then modified the LIF (β = 0.0001) and Synaptic neuron models' hyperparameters (either β_*syn*_ = 0.0001 or α_*syn*_ = 0.0001 or both) to systematically induce a hyperexcitability-like regime and evaluate its impact on network dynamics and reconstruction performance. Due to computational constraints associated with full-scale SNN training on the MNIST dataset, the majority of experiments were conducted using a single-run configuration, consistent with the study's objective of providing a mechanistic analysis rather than performance optimization. To assess the robustness of the key finding regarding noise-driven compensation, additional experiments evaluating test MSE across noise levels were repeated using three independent random seeds (123, and 999). Results from this analysis are reported as mean ± standard deviation across runs. Training was performed using surrogate gradient learning with an arctangent surrogate function, surrogate.atan(α = 2.0). All other hyperparameters, including optimizer settings and training configuration, were kept fixed across experiments to isolate the effect of excitability and noise.

We define hyperexcitable-like regimes by parameter configurations corresponding to increased effective conductivity (i.e., reduced β and/or altered synaptic decay), and subsequently evaluate their impact on reconstruction performance. Among the explored parameter configurations, the regime producing the strongest degradation in reconstruction performance is used as a representative hyperexcitable-like condition.

We computed the lossy information encoding and reconstruction performance (*MSE* and *MSE*_*d*_) for five scenarios, including the benchmark and overfiring-like cases; see Table 2. For this analysis, we split the MNIST dataset into 60, 000 digits for training and 10, 000 for testing, all images were resized to have 32 × 32 pixels, converted to grayscale, and normalized to ensure uniform data formatting (aligning well with the proposed SNNs). We also set the threshold value τ = 1 and set a time step of 5 ms for each simulation run; split the data set into batches of equal size (250, in this case) and used 50 epochs, resulting in 12, 000 and 2, 000 iterations for training and testing, respectively. Neuron models were imported from the installed *PyTorch* and *snntorch* libraries and the required neuron models were called using “*snn.leaky()*” and “*snn.synaptic()*” commands. For the benchmark case (scenario 1), the obtained *MSE* shows a high similarity between the input and output data processed by the autoencoder (when using natural-based hyperparameter values), while the *MSE*_*d*_ demonstrate a fast convergence to a constant error performance (that is, low *MSE* with a low number of iterations). In Scenario 2, it seems that there is not a huge difference in loss values compared to Scenario 1, which could be associated with the network topology and architecture, as the synaptic neuron models perform without a change in their hyperparameters, as we had in Scenario 1. By modifying β_*syn*_, we can excite neurons to excessively fire action potentials and to be less sensitive to input changes, impairing the ability of the network to process information properly (instability and loss of information). This issue can be seen in Scenario 3, where we obtained the worst performance (highest *MSE* and *MSE*_*d*_ values), thus becoming our overfiring-like scenario. In contrast, we obtained the best performance in Scenario 4, where we only modified α_*syn*_. By keeping β_*syn*_ unchanged while modifying α_*syn*_, neurons remained sensitive to input data, maintaining a balance between excitatory and inhibitory signals, and improving overall system performance. When modifying both α_*syn*_ and β_*syn*_ (Scenario 5), we obtained *MSE* and *MSE*_*d*_ performances similar to Scenario 3, which showed that reducing α_*syn*_ did not counteract the effect of a slow decay rate for the synaptic neuron model.

**Table 2:** Sample results of different scenarios based on the MSE loss value of the model.

Scenario	Sub-scenario	*MSE*	*MSE*_*d*_
1 - Benchmark	No changes	0.099	635.32
2 - LIF	β = 0.0001	0.076	473.96
**3 - Synaptic**	**β_*syn*_ = 0.0001**	**0.277**	**1396.8**
4 - Synaptic	α_*syn*_ = 0.0001	0.064	504.54
5 - Synaptic	α_*syn*_ = 0.0001, β_*syn*_ = 0.0001	0.10	1098.64

The overfiringlike scenario is highlighted in bold.

**Figure 5:**
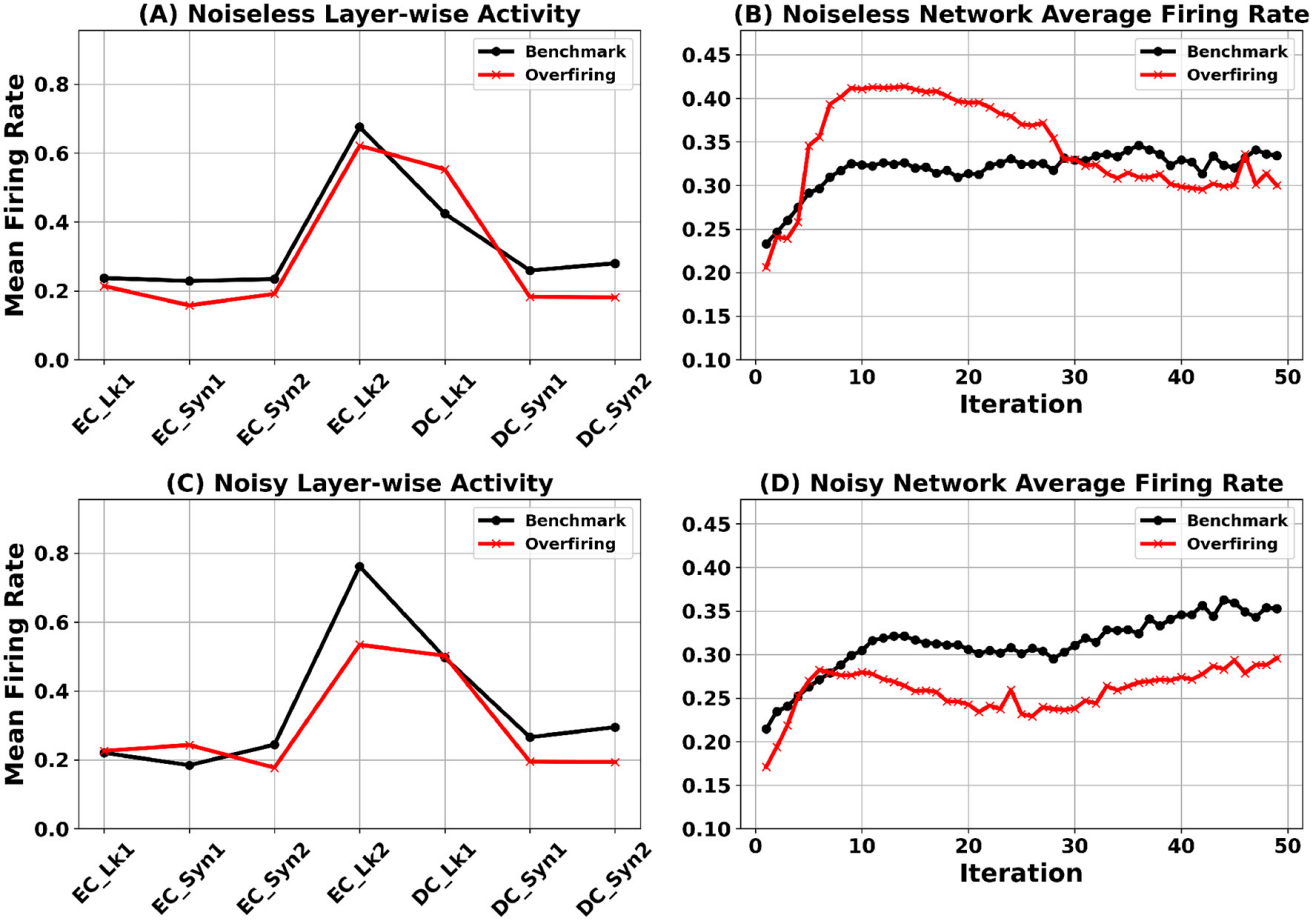
Average firing rate of **(A)** Noiseless (**top**) vs. **(C)** Noisy (**bottom**) data across SNN layers in the final epoch, and **(B,D)** across iterations for both benchmark (black) and overfiring-like (red) regimes.

Figure 5A,C shows the final-epoch mean firing rates across encoder and decoder layers.

In the noiseless condition (Figure 5A), encoder layers consistently exhibit reduced firing rates under the hyperexcitability-like regime (e.g., EC_Syn1: 0.2288 → 0.1577; EC_Syn2: 0.2339 → 0.1913), indicating impaired temporal integration due to faster membrane decay (reduced membrane decay rate). In contrast, decoder layers exhibit heterogeneous behavior: the first decoder leaky layer shows increased activity (DC_Lk1: 0.4242 → 0.5525), while downstream synaptic layers display reduced firing (DC_Syn1: 0.2591 → 0.1824; DC_Syn2: 0.2800 → 0.1811). This non-uniform pattern demonstrates that the hyperexcitability-like regime does not correspond to a global increase in firing activity, but rather induces a redistribution of activity across layers.

Under the noisy condition (Figure 5C), the interaction between input perturbations and reduced temporal integration further reshapes this activity distribution. Certain encoder layers exhibit increased firing rates under the hyperexcitability-like regime (e.g., EC_Syn1: 0.1846 → 0.2431), suggesting partial restoration of responsiveness. However, deeper encoder and decoder layers show reduced activity (e.g., EC_Lk2: 0.7619 → 0.5341), indicating impaired propagation of information.

These results suggest that noise does not uniformly amplify activity, but instead redistributes firing in a manner that partially compensates for instability induced by reduced temporal integration.

Figure 5B shows the temporal evolution of network-wide firing rates. The hyperexcitability-like regime exhibits a non-monotonic profile, characterized by elevated activity during early training epochs followed by a progressive decline below the benchmark condition. This contrasts with the relatively stable firing profile observed in the benchmark regime and indicates reduced temporal stability.

Under the noisy condition (Figure 5D), the hyperexcitability-like regime exhibits consistently lower average firing rates compared to the benchmark, suggesting that noise further limits effective temporal integration at the network level.

**Figure 6:**
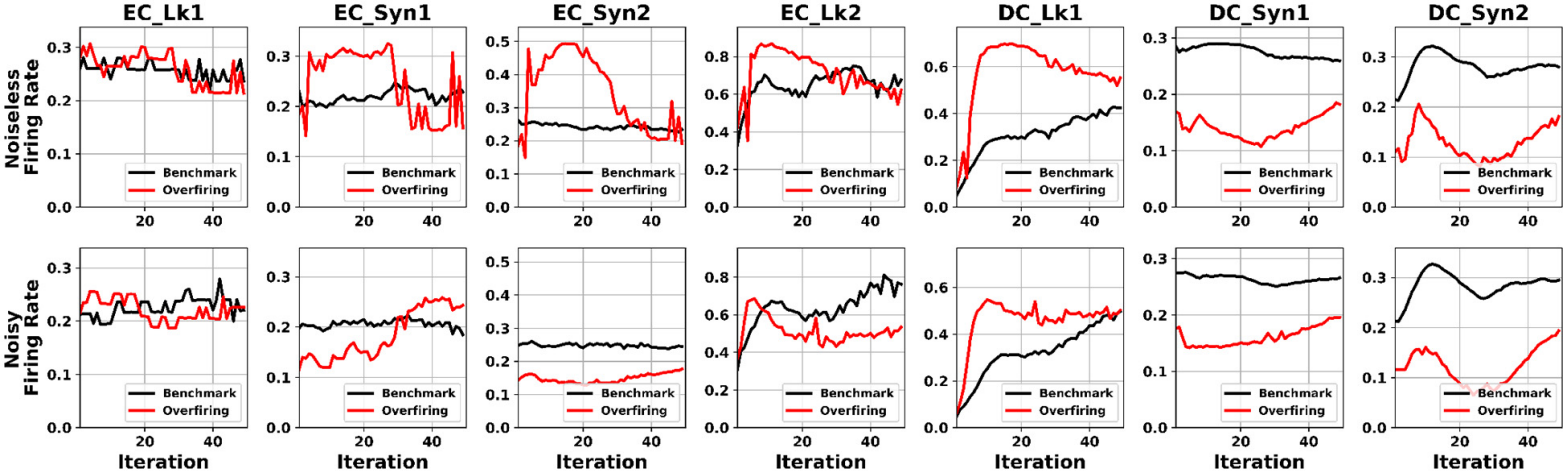
Encoder and Decoder full layers' firing rate in noiseless (**top row**) and noisy (**bottom row**) conditions for benchmark (black curves) vs. overfiring-like (red curves) regimes. Notably, the overfiring-like regime does not result in uniformly increased activity, but instead induces a redistribution of firing across layers, with suppressed encoder activity and elevated decoder responses.

Figure 6 provides a detailed layer-wise comparison of firing rates across all conditions. Consistent with the aggregate analysis, the hyperexcitability-like regime is characterized by a redistribution of activity rather than a uniform increase in firing. Specifically, encoder synaptic layers show reduced activity, while decoder leaky layers exhibit increased activation, indicating an imbalance in signal propagation across the network. Importantly, the introduction of noise partially mitigates this imbalance by restoring activity in previously suppressed layers (e.g., EC_Syn1) while reducing excessive activation in dominant layers (e.g., EC_Lk2), supporting the interpretation that noise can stabilize spike-based information flow under reduced temporal integration.

Overall, these findings indicate that hyperexcitability-like dynamics in the proposed SNN are better characterized by instability in activity distribution rather than an overall increase in firing rate.

### 3.1 Image compression performance analysis

We compressed a set of noisy and noiseless handwritten digit images based on our definitions of benchmark and overfiring-like scenarios with the same simulation setup from our previous analysis. For our noisy image analysis, we added Gaussian noise only to the training set of MNIST handwritten digit images before processing them through the proposed SNN-based autoencoder.

**Figure 7:**
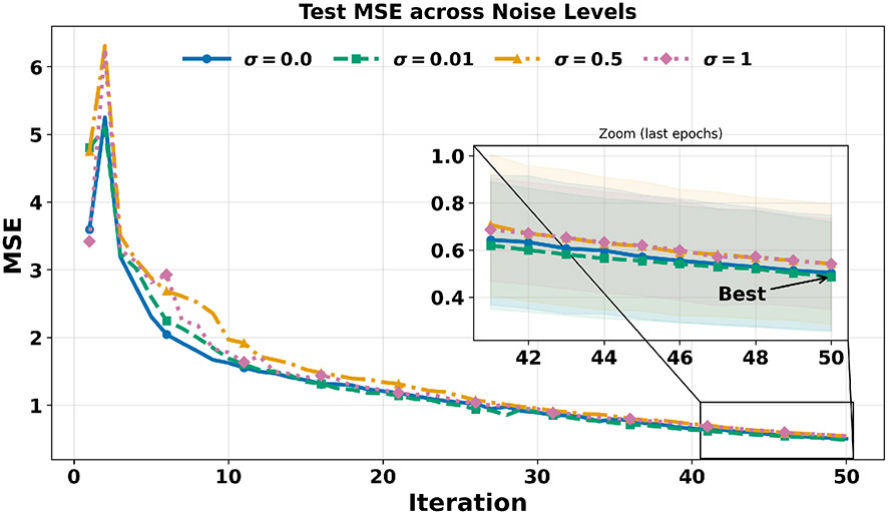
Mean Squared Error (MSE) curve of testing data representing four different noise level (σ = 0, σ = 0.01, σ = 0.5, σ = 1.0). Full iterations plus the magnitute view of the final iterations are shown to show the performance difference.

Figure 7 presents the test MSE across four noise levels (σ = 0, 0.01, 0.5, 1.0), evaluated over 50 iterations and averaged across three independent random seeds ([42, 123, 999]). Results are reported as mean ± standard deviation to capture variability across stochastic initializations. A non-monotonic relationship between noise intensity and reconstruction performance is observed. Specifically, moderate noise (σ = 0.01) consistently yields the lowest mean test error, whereas both the noiseless condition (σ = 0) and higher noise levels (σ≥0.5) result in degraded performance. To facilitate interpretation, the full trajectory and a magnified view of the final iterations are presented, highlighting the performance differences between noise conditions during different stages of learning.

Quantitatively, σ = 0.01 achieves the lowest mean test error (0.4864 ± 0.2325). Notably, the best individual run attains a substantially lower error (0.1712), indicating that moderate noise can enable improved optimization trajectories, albeit with variability across random seeds. These results suggest that controlled noise injection can enhance learning dynamics, while excessive noise might disrupt signal fidelity. This supports the interpretation that moderate noise acts as a stabilizing factor in training, rather than a purely degradative perturbation.

**Figure 8:**
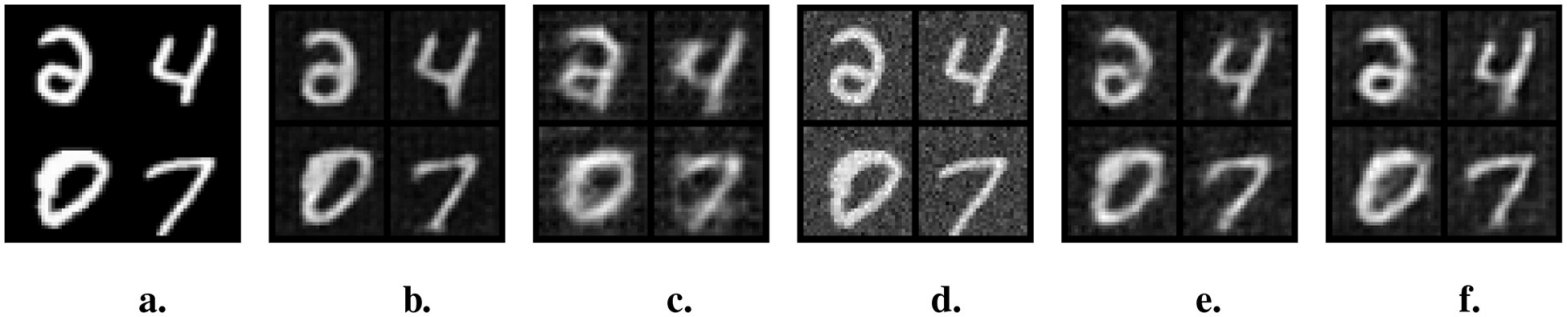
Example of handwritten digit image from MNIST and its reconstructed images for the benchmark and overfiring-like scenarios for noiseless and noisely input data. **(a)** Noiseless input digits fed to the proposed system. **(b)** Reconstructed digits from noiseless input data for the benchmark scenario. **(c)** Noiseless reconstructed digits from noiseless input data for the overfiring-like scenario. **(d)** Noisy input data fed to the proposed system. **(e)** Reconstructed digits from noisy input data for the benchmark scenario. **(f)** Reconstructed digits from noisy input data for the overfiring-like scenario.

Please note that Gaussian noise introduces stochastic perturbations analogous to background neural variability that can generate fluctuations in the values that represent the colors / gradients of the image pixels (Buades et al., 2005). The compressed images obtained as output of our proposed SNN-based autoencoder are shown in Figure 8. When the input data are not subjected to any noise (see Figure 8a–c), the compressed image shows visible data loss (i.e., reduction of image fidelity) for both scenarios, especially when the SNN-based autoencoder is operating under overfiring-like conditions (worst case)(hyperexcitable-like regime exhibiting unstable network dynamics). When compressing noisy images considering a zero mean μ = 0 and small variance σ = 0.01 Gaussian noise (see Figures 8d–f), the overfiring-like scenario produced slightly better quality output images than the benchmark case, in contrast to compressing noiseless images.

**Figure 9:**
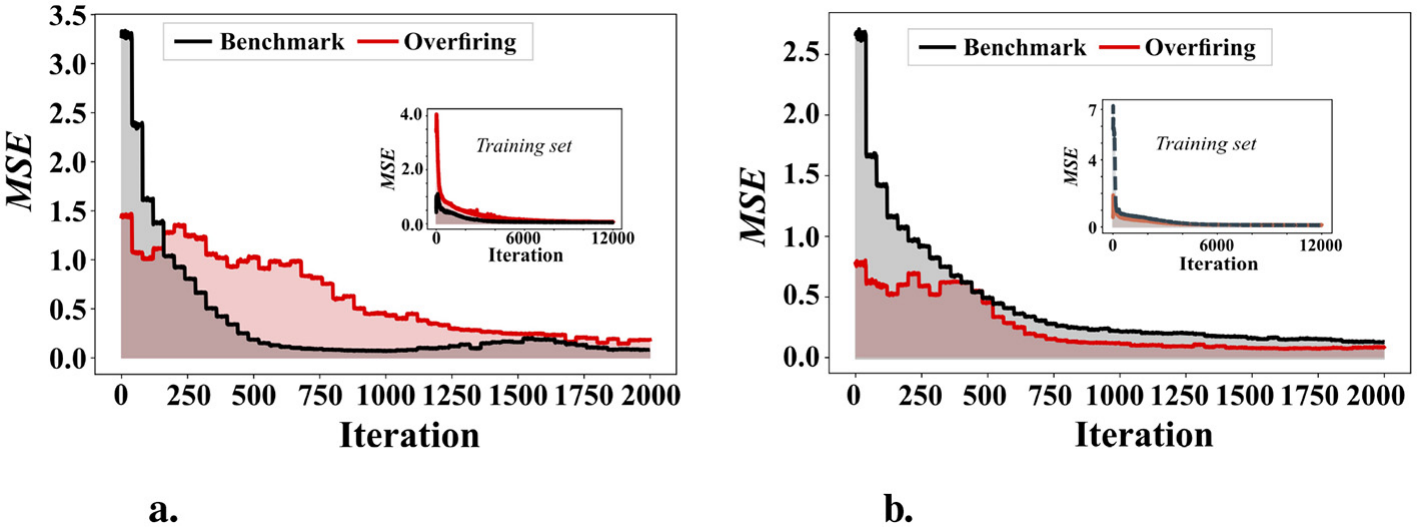
*MSE* comparison plots for the training and testing datasets (benchmark and overfiring-like scenarios). **(a)** *MSE* for the noiseless case. **(b)** *MSE* for the noisy case.

Next, we computed *MSE* and *MSE*_*d*_ to better understand the results shown in Figure 8 and plotted the result in Figure 9. The noiseless plot *MSE* (Figure 9a) shows an exponential reduction in image reconstruction error for the benchmark scenario, which also has a lower *MSE* than in the overfiring-like case. Figure 9a also shows that *MSE* converges to similar values for both scenarios despite having different convergence rates. This means that when the SNN-based autoencoder is performing under a excessive spiking rate, it requires prolonged exposure to stabilize learning dynamic how to compress the handwritten image with a visual fidelity similar to the benchmark case. Quantitatively, the obtained *MSE*_*d*_ for the overfiring-like scenario is 68% higher than the benchmark case (indicative of impaired learning under hyperexcitable-like dynamics), see Table 3. When compressing noisy images (see Figure 9b), the SNN-based autoencoder with the benchmark configuration produced a higher *MSE* than the overfiring-like case, requiring less data to converge to a lower reconstruction error (the benchmark required 375 batches to achieve the same *MSE* as in the overfiring-like scenario), which was only matched by the benchmark scenario at the end of our simulation. In this case, the *MSE*_*d*_ obtained for the overfiring-like scenario was 44% lower than in the benchmark case, as shown in Table 3, and these results support a noise-driven compensatory mechanism from Figure 8.

**Table 3:** *MSE*_*d*_ performance comparison for benchmark and overfiring-like scenarios, for a with/without noise on the input data (evaluated from Figures 8a, b.)

**Metric**	**Input data**	**Benchmark (*A*)**	**Overfiring-like (*B*)**	**Diff. $\left(\frac{B-A}{A}\right)$**
*MSE*_*d*_	w/o Noise	719.7	1,210.1	68%
	Noisy	862.4	484.6	−44%

### 3.2 Autoencoder's layers learning performance analysis

**Figure 10:**
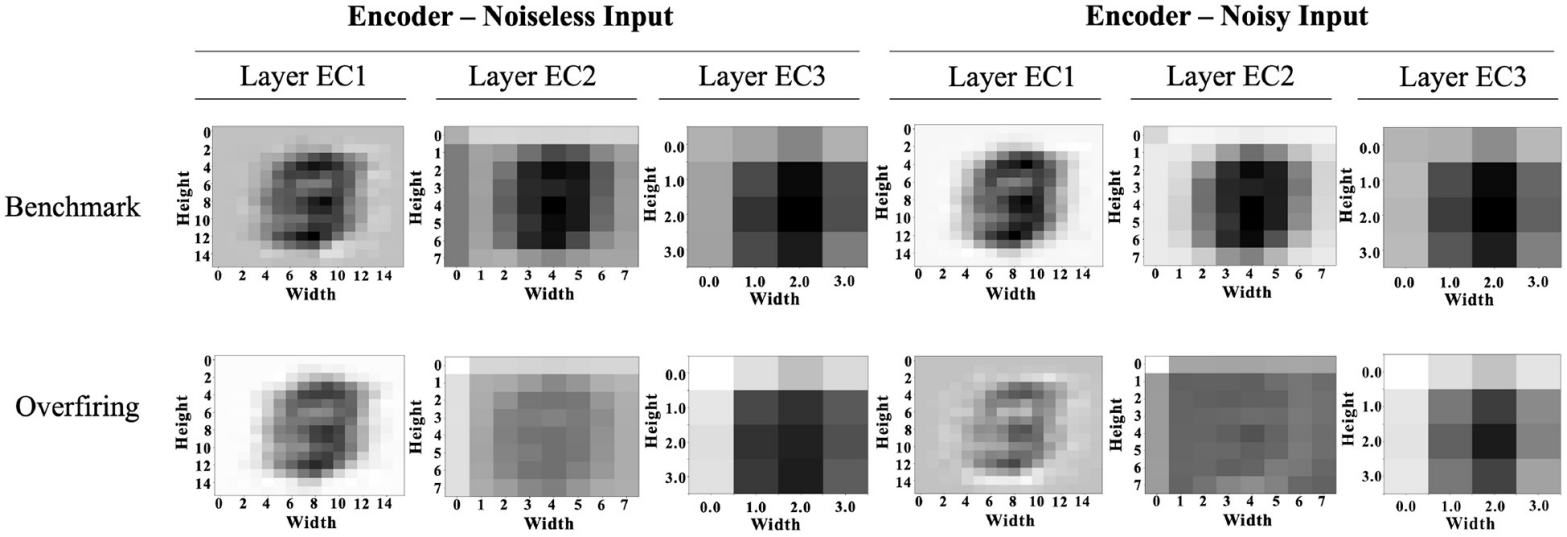
Visualization of Encoder intermediate layers activity (EC1–EC3) under benchmark and overfiring-like conditions, for noiseless and noisy inputs.

**Figure 11:**
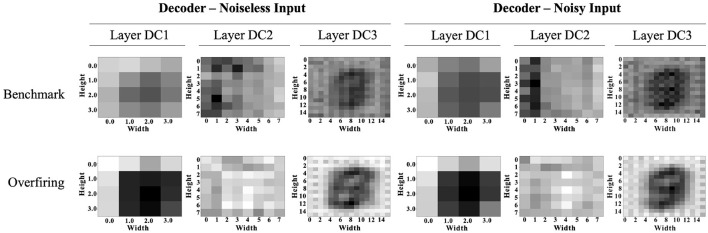
Visualization of Decoder intermediate layers activity (DC1–DC3) under benchmark and overfiring-like conditions, for noiseless and noisy inputs.

In our previous analysis, we observed the learning performance based on *MSE* and *MSE*_*d*_ calculated on the images output from the autoenconder. We further investigate the learning performance of the proposed architecture by extracting the feature maps from each autoencoder's layers and computing the *MSE*_*l*_. Please note that we ran our simulations using the same simulation setup as in previous analyses and did not consider any additional noise in the layers. Figure 10, Figure 11 show the images extracted from each encoding and decoding layer, where we can observe the damage caused by the high spiking rate in the overfiring-like scenario compared to the benchmark on the compressed image data. In particular, the image pixels around the digits in layer EC1 were lighter for the overfiring-like scenario than for the benchmark. This effect propagated through the remaining layers, affecting both the outer and inner pixels processed in the encoding layers; see Figure 10. When adding Gaussian noise, the system produces the opposite behavior. In this case, the excess of spikes is countered by Gaussian noise (affecting the ratio between lighter and darker pixels in the feature maps), resulting in improved performance for the overfiring-like scenario, and the produced results are visually similar to the benchmark (for the noiseless case); see Figure 10. Next, we observe the image data processed in the decoder layers; see Figure 11. For the noiseless case, the images extracted from the decoder have a higher occurrence of lighter pixels in the overfiring-like scenario than the benchmark, resulting in a misrepresentation of the reconstructed image data on each decoding layer. Furthermore, the overfiring-like scenario shows a distinguishable visual difference in comparison to the benchmark, which could be correlated to aggregation of damaged features (i.e., composition of the damage suffered in all previous layers) combined with the higher conductivity of the decoding layers. When applying Gaussian noise, the first layer of the decoder produces the most damaged feature map of all scenarios. This effect is explained by how the noise affects the latent space (explored in our next analysis), resulting in a performance reduction of the first decoder layer. Despite that, the remaining layers (DC2 and DC3) produced visually better images than the noiseless case, and the final feature map is more visually similar to the original input handwritten digit image in this scenario.

We quantified the visual results obtained in our analysis of the autoencoder layers using *MSE*_*l*_; see Table 4. The difference between noisy and noiseless *MSE*_*l*_ for layers EC1 and EC2 was quite similar, suggesting a low impact of Gaussian noise in these layers. However, layers EC3, DC1, and DC2 showed a great reduction in *MSE*_*l*_ between the noisy and noiseless cases, including a smaller reconstruction error for layer DC1 when compressing noisy image data. It is important to note that the average *MSE*_*l*_ for the noiseless case is slightly smaller than for the noisy case (0.2766 and 0.2996, respectively), suggesting that while the noise effect can be observed in each layer, it produces minor quantitative changes in the autoencoder as a whole. Moreover, we can also observe from Table 4 that for both types of inputs, *MSE*_*l*_ is lower for the encoder layers (EC1, EC2, and EC3) compared to the decoder layers (DC1, DC2, and DC3), suggesting that the latent space damages the compressed images (and constitute a critical bottleneck for information stability).

**Table 4:** *MSE*_*l*_ performance comparison for a with/without noise on the input data.

**Layers**	MSE_l_		
	**Noiseless input (*C*)**	**Noisy input (*D*)**	**Diff. $\left(\frac{D-C}{C}\right)$**
Layer EC1	0.2003	0.2546	27.11%
Layer EC2	0.2107	0.2676	27.01%
Layer EC3	0.2388	0.2393	0.21%
Layer DC1	0.3838	0.3687	−3.93%
Layer DC2	0.3445	0.3550	3.05%
Layer DC3	0.2817	0.3125	10.93%

**Figure 12:**
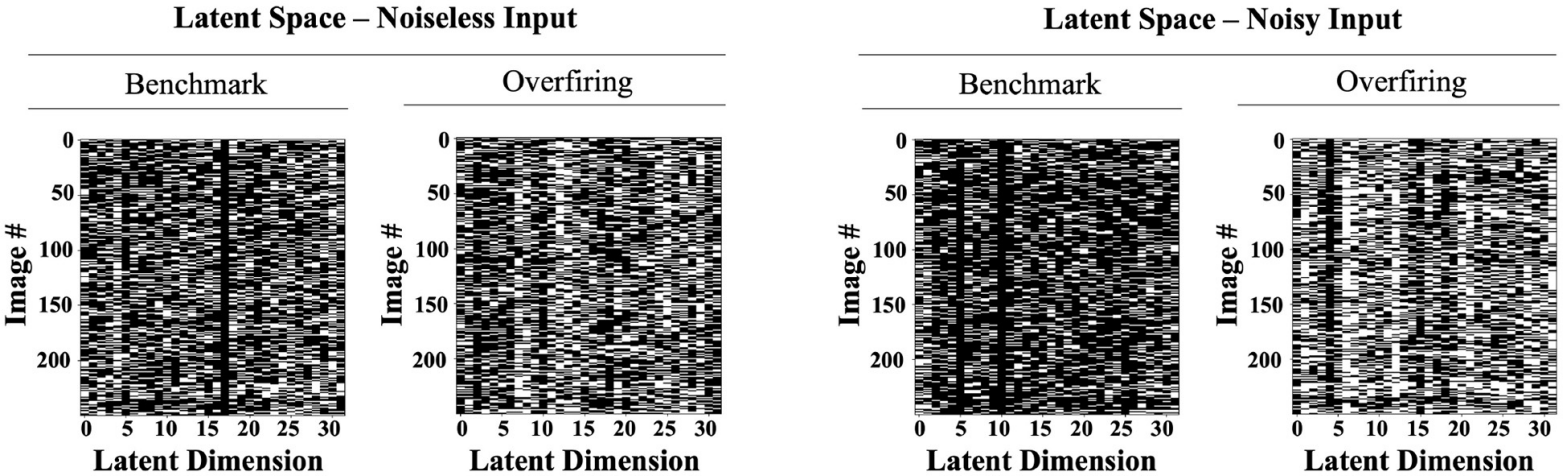
Spike activation raster plots extracted from the latent space for both benchmark and overfiring-like states under various conditions. The latent space represents a compressed version of the input image data, capturing essential features and patterns. Each plot shows the spike activation for 250 images across 32 latent dimensions, where black indicates an active spike and white indicates the opposite.

Our results from the encoder and decoder layers led us to investigate the autoencoder's latent space, where the image data abstract features and patterns captured by the SNN's learning process are represented in a compact form. Figure 12 shows the output of the latent spaces for all images in a batch, for both scenarios, and separated from the other layers. Here, the latent space was composed by 32 dimensions, and we used a raster plot to show the spike activation of each latent dimension. From Figure 12, we can observe that some latent dimensions are activated more frequently in all images, indicating that they capture some prevalent and significant patterns in the data. For the noiseless case, the latent dimension activation was reflecting unstable population dynamics under hyperexcitability (overfiring-like), where the SNNs had more problems identifying the relevant information from the original images. When adding Gaussian noise, more latent dimensions were activated than in the noiseless case for the benchmark scenario, worsening the effect of the spiking rate on the activation of latent dimensions. On the other hand, less latent dimensions were activated in the overfiring-like scenario, when compared to the noiseless case, suggesting a noise-driven attenuation of pathological excitability on the SNNs by the Gaussian noise. Together, these findings demonstrate how noise-driven compensation can interact instability in learning and information processing in otherwise unstable hyperexcitable-like network regimes, motivating the mechanistic interpretation developed in the Discussion.

## 4 Discussion

This study illustrates how SNNs can be used not merely as computational tools but as experimental proxies for biological networks. By parameterizing neuronal and synaptic dynamics, we model a hyperexcitability-like regime as a computational abstraction of altered excitability, and investigate the effect of controlled perturbations on network behavior. The observation that Gaussian noise restores function highlights how SNNs can serve as tractable in silico laboratories, where hypotheses about abnormal brain dynamics and interventions can be rapidly tested. Importantly, the objective of this study is not to optimize reconstruction performance, but to use SNNs as a controlled framework for analyzing how dynamical perturbations affect spike-based information processing.

Throughout our analysis, we observed that an increased spiking rate can damage the SNN learning process required to compress images with a low error. In particular, the Synaptic neuron model with a decay rate much smaller than the typical value (see Scenario 3 in Table 2) resulted in the highest error, indicating more damage and impaired learning during the overfiring-like condition with this parameter. This poor performance aligns with the characteristics of epileptic conditions, where abnormal synaptic activity, particularly involving ionotropic glutamate receptors such as NMDA and AMPA, leads to neuronal hyperexcitability and disrupted information processing. However, we emphasize that this correspondence is qualitative rather than mechanistic. The higher error observed in this scenario may reflect the detrimental effects of excessive synaptic activity, similar to the pathological synaptic changes seen in temporal lobe epilepsy (TLE). In TLE, increased neurotransmitter release and altered receptor sensitivity contribute to recurrent seizures and instability of the neural network. Accordingly, the synaptic neuron model provides a useful computational abstraction for studying how altered excitability may affect spike-based information processing, rather than a direct biophysical replication of epileptic dynamics.

The proposed SNN model, provides a simplified and controlled framework in which key qualitative properties of excitability can be studied. Therefore, the term “hyperexcitability-like regime” is used to denote a computational abstraction rather than a direct physiological model. The apparent discrepancy between biophysical hyperexcitability (increased firing rates with higher *g*_*Na*_ or *g*_*l*_) and the reduced steady-state firing observed in the SNN overfiring-like regime highlights a fundamental distinction between conductance-based neurons and simplified spiking neuron models. In conductance-based models, increased ion channel conductivity enhances excitability without directly limiting temporal integration. In contrast, reducing β in LIF/Synaptic models increases instantaneous responsiveness while simultaneously shortening the effective membrane time constant, thereby impairing sustained signal propagation. Thus, reduced β induces a hyperexcitability-like regime characterized not by sustained high firing rates, but by transient overactivation followed by reduced effective signal propagation. This interpretation is further supported by the layer-wise firing rate analysis (Figure 5), which reveals that the overfiring-like regime does not produce uniformly elevated activity. Instead, it results in heterogeneous activity redistribution across layers, with increased activation in decoder layers (e.g., Dec_Lk1) and reduced activity in synaptic encoding layers. This spatial imbalance is consistent with the quantitative firing rate analysis, where decoder layers (e.g., Dec_Lk1) exhibit elevated activity while synaptic encoding layers show suppressed firing, confirming that hyperexcitability-like dynamics disrupt coordinated signal propagation rather than uniformly increasing activity. Figure 6 provides a detailed layer-wise comparison under noiseless and noisy conditions, enabling direct visualization of these effects.

When compressing images, the error performance of a neural network is expected to improve when more data is processed. We could observe this effect in Figure 9a where *MSE* performance is improved for a higher number of iterations (both benchmark and overfiring-like scenarios). It was also expected that the learning performance for compressing noisy images would be reduced; however, when compressing noisy images with an overfiring-like SNN we can obtain a lower error with a lesser amount of input data, resulting in an improved learning performance when compared with a SNN operating under typical conditions (benchmark scenario). Furthermore, the noisy image compression SNN also benefit from processing more data to obtain a smaller *MSE*, see Figure 9b. Based on this result, we can infer that the Gaussian noise helped the SNN-based autoencoder quickly learn to compress the image with a visual fidelity similar to the original noiseless MNIST handwritten digit data. In particular, when no noise is applied, the SNN-based autoencoder needed around 1,500 iterations to achieve a similar *MSE* value for the benchmark and overfiring-like cases, while 1/3 of that number was required when compressing noisy images. Therefore, Figure 9b quantified and confirmed the results shown in Figure 8, since the overfiring-like *MSE*_*d*_ was 44% lower than the one observed for the benchmark scenario, indicating that Gaussian noise injection improved the autoencoder's learning performance; see Table 3. The observed noise-driven compensation effect may arise from three interacting mechanisms: (i) input perturbations that prevent premature convergence to suboptimal attractors, (ii) implicit regularization that stabilizes learning dynamics, and (iii) modulation of spike timing variability, which improves information propagation under reduced temporal integration.

The unexpected result of noisy image compression by an overfiring-like SNN, led us to investigate the outputs of each layer of the proposed autoencoder in an attempt to understand how the noise is affecting the learning performance of our proposed SNN architecture. The feature maps obtained from the intermediate layers of the autoencoder shown in Figure 10, Figure 11 have a higher number of white pixels in their outer region for the overfiring-like scenario. These lighter pixels resulted from the excess of spikes, indicating a destructive interference on each image composition, which affects the autoencoder's capability of extracting the required image features to reconstruct the digits with a higher visual fidelity. However, when we compressed noisy images, the interference observed in the noiseless case was countered, and the autoencoder operating in the overfiring-like scenario became able to reconstruct the input image with a higher visual fidelity than in the benchmark scenario. The expected fluctuations on the gray gradients of the handwritten digit image caused by Gaussian noise directly contribute to the improvement of the learning performance of our autoencoder. Finally, we could observe that the minimum *MSE* was found in layer EC1, while the maximum *MSE* was found in layer DC1, indicating the damage due to the compression of the image in the latent space (validated by the high randomness observed in Figure 12), which cannot be fully restored in the decoding layers, and should be targeted in future works to improve the overall learning performance of the autoencoder.

The layer-wise results in Figure 5, Figure 6 demonstrate that the hyperexcitability-like regime is characterized by a redistribution of activity rather than a global increase in firing rate. Reduced β enhances instantaneous responsiveness while limiting temporal integration, leading to suppressed activity in early encoding stages and unstable propagation across deeper layers. The introduction of noise partially compensates for this imbalance by reactivating underactive layers while reducing excessive activation in downstream stages, resulting in a more balanced activity profile across the network. These findings indicate that hyperexcitability-like dynamics in SNNs are better described as an instability in activity distribution rather than a uniform increase in firing rates. The resulting imbalance leads to inefficient information propagation, where excessive activation in decoder layers coexists with reduced effective transmission in earlier encoding stages.

The stabilizing effect of noise suggests that controlled perturbations can partially restore functional balance by enhancing temporal variability and preventing convergence to inefficient activity regimes.

More broadly, our work contributes to the integration of artificial and biological network models in systems neuroscience. Unlike conventional artificial neural networks (ANNs), SNNs explicitly model temporal dynamics and spike-based signaling, making them particularly suitable for studying activity-dependent phenomena such as hyperexcitability-like regimes. Whereas ANNs capture high-level computational principles and detailed biophysical models (like HH) capture cellular dynamics, SNNs occupy a middle ground: sufficiently abstract for scalable experiments, yet grounded in biologically meaningful parameters. By demonstrating how pathological states and interventions/compensations can be emulated in SNNs, we provide a framework for bridging ANN research with systems neuroscience, aligning with the goal of unraveling neural network dynamics across artificial and biological domains.

## 5 Conclusion

In this work we employed spiking neural networks (SNNs) as *biological-related network analogs* and tractable *testbeds* to investigate how altered neuronal and Synaptic dynamics affect network computation and learning. Using an autoencoder built from Leaky Integrate-and-Fire and synaptic neuron models, we parametrically induced a hyperexcitability-like regime to emulate hyperexcitability analogous to NaV channel dysfunction in hippocampal circuits. Under these pathological parameter regimes, the SNN exhibited degraded information encoding and reconstruction performance, consistent with impaired processing in hyperexcitable biological networks. Crucially, introducing controlled Gaussian noise into the input image data partially restored reconstruction quality and improved learning metrics, indicating that stochastic modulation can counteract instability otherwise unstable network regimes.

Importantly, the observed hyperexcitability-like regime is characterized not by sustained increases in firing rates, but by transient overactivation and unstable signal propagation across layers, as evidenced by the layer-wise firing rate analysis.

Together, these findings demonstrate that (1) specific SNN parameter regimes can reproduce qualitative signatures of hyperexcitability-like dynamics, and (2) noise-driven compensation emerges as a plausible explanatory mechanism for mitigating functional breakdown in such regimes. Beyond improving SNN robustness for engineering tasks, our results position spiking autoencoders as scalable, biologically grounded platforms for hypothesis-driven studies of neural dysfunction and candidate interventions. This approach bridges ANN methodologies with mechanistic models in systems neuroscience and provides a concrete pathway for using hybrid computational frameworks to generate testable hypotheses about how altered excitability and stochastic perturbations influence network-level information processing.

## Data Availability

The raw data supporting the conclusions of this article will be made available by the authors, without undue reservation.
